# Clinical application of the modified posterolateral approach for treating posterior tibial plateau fractures

**DOI:** 10.3389/fbioe.2023.1150541

**Published:** 2023-02-17

**Authors:** Wang Shuaishuai, Zhang Minglei, Yu Yue, Wang Dapeng, Zhu Tongtong, Liu Huimin

**Affiliations:** ^1^ Department of Orthopedics, China-Japan Union Hospital of Jilin University, Changchun, China; ^2^ Department of Orthopedics, Siping Central Hospital, Siping, China; ^3^ Mengzhou Fuxing Hospital, Meng Zhou, China

**Keywords:** tibial plateau, modified posterior lateral approach, posterior tibial plateau fracture, collapse of the articular surface, internal fixation, complications

## Abstract

**Objective:** To investigate the therapeutic efficacy of the modified posterolateral approach on tibial plateau fractures.

**Methods:** Forty-four patients with tibial plateau fractures were enrolled in the study and divided into two groups—control and observation—according to the different surgical procedures. The control group underwent fracture reduction *via* the conventional lateral approach, while the observation group underwent fracture reduction *via* the modified posterolateral strategy. The depth of tibial plateau collapse, active mobility, and the Hospital for Special Surgery (HSS) score and Lysholm score of the knee joint at 12 months after surgery were assessed in comparison to the two groups.

**Results:** The amount of blood loss (*p* < 0.01), duration of surgery (*p* < 0.05), and depth of tibial plateau collapse (*p* < 0.001) were significantly less in the observation group compared with the control group. In addition, compared with the control group, the observation group exhibited significantly better knee flexion and extension function and significantly higher HSS and Lysholm scores at 12 months after surgery (*p* < 0.05).

**Conclusion:** The modified posterolateral approach for posterior tibial plateau fractures has less intraoperative bleeding and a shorter operative time compared with the conventional lateral approach. It also effectively prevents postoperative tibial plateau joint surface loss and collapse, promotes the recovery of knee function, and has few postoperative complications and good clinical efficacy. Thus, the modified approach is worth promoting in clinical practice.

## 1 Introduction

Tibial plateau fractures are a common fracture that involves the joint surface and therefore require high-level anatomic reduction. The joint surface of the upper tibia has a posterior slope to the tibial stem, and this posterior slope provides the anatomical basis for posterior tibial plateau fractures (Jiwanlal and Jeray, 2016; van den Berg et al., 2017; van den Berg et al., 2020). During flexion of the knee joint, axial violence acts on the knee joint, causing the femoral condyle to strike the posterior tibial plateau and resulting in fracture of the posterior tibial plateau on the coronal plane. Yang et al. (2013); Wang et al. (2016) concluded that the incidence of posterior tibial plateau fractures is approximately 28%. Improper management of posterior tibial plateau fractures can seriously affect the function of the lower limb in patients, leading to complications such as pain and deformity and marked impacts on quality of life. Surgical treatments for displaced posterior tibial plateau fractures aim to restore the flatness of the joint surface, joint stability, and normal force lines through open reduction and internal fixation (Lin et al., 2015). The quality of reduction and fixation acts as a determinant of surgical outcome, and the commonly used surgical approaches are anterolateral approach, posteromedial approach, and posterolateral approach (Luo et al., 2010; Zu et al., 2020; Giordano et al., 2022). There are more types of postero-lateral approaches reported, which can be divided into two categories: osteotomized and non-osteotomized. These approaches allow for fracture reduction and fixation through the opening of a larger skin tissue flap. However, such opening subsequently impacts postoperative wound healing, easily injures important blood vessels and nerves in the popliteal fossa and causes greater soft tissue loss, and is not conducive to postoperative recovery and incision healing (Solomon et al., 2010). Good joint surface reduction and stable fixation of the fracture combined with early functional exercises can effectively reduce damage to the knee function of patients with posterior tibial plateau fractures (Lin et al., 2015). The knee joint is a weight-bearing joint with the highest functional requirements and activity frequency in the body. Therefore, functional exercises need to be performed at the earliest opportunity after surgery. However, early functional exercises may increase the risk of joint surface collapse of the tibial plateau. Postoperative collapse of the tibial plateau can seriously affect the daily life of patients and may even require surgical treatment (if the joint surface is depressed >2 mm). Consequently, it is crucial to monitor for and prevent postoperative tibial plateau collapse (Shen et al., 2021; Ming et al., 2022). Compared with other posterior lateral approaches, we adjusted the position to prone to make the incision smaller and more medial, to reduce soft tissue injury and to avoid overstretching the common peroneal nerve.

This study aimed to address the potential problems encountered during surgical treatment of posterolateral tibial plateau fractures by evaluating the use of a modified posterolateral approach with bone plate internal fixation *via* a smaller incision.

## 2 Subjects and methods

### 2.1 Case presentation

A total of 44 patients (29 males and 15 females) with posterolateral tibial plateau fractures were enrolled from September 2018 to June 2022 at the Department of Traumatology and Orthopedics. The patients were divided into two groups depending on the surgical treatment: 19 patients were undergoing surgical treatment *via* the modified posterolateral approach (observation group) and 25 patients were undergoing surgical treatment *via* the conventional lateral approach (control group) (Table 1). The method was approved by the Ethics Committee of China-Japan Union Hospital.

**TABLE 1:** Baseline data of enrolled patients.

Patient no.	Sex (M/F)	Age (years)	Three-column fracture classification	Preoperative preparation (days)	Study group
1	M	46	Posterior	6	O
2	F	52	Medial (combined)	8	O
3	M	23	Three-column	8	O
4	M	40	Posterior (combined)	7	O
5	F	48	Posterior	3	O
6	M	38	Posterior (combined)	7	O
7	M	27	Posterior	7	O
8	M	46	Three-column	7	O
9	M	57	Posterior	7	O
10	F	51	Three-column	10	O
11	M	42	Posterior	7	O
12	F	46	Posterior (combined)	7	O
13	F	39	Posterior (combined)	8	O
14	M	34	Medial (combined)	7	O
15	M	40	Posterior	7	O
16	M	47	Three-column	14	O
17	M	65	Posterior (combined)	7	O
18	F	36	Three-column	7	O
19	M	59	Posterior (combined)	6	O
20	M	46	Posterior (combined)	4	C
21	F	52	Posterior (combined)	8	C
22	M	28	Three-column	8	C
23	M	50	Posterior (combined)	7	C
24	F	48	Posterior	3	C
25	M	38	Posterior (combined)	7	C
26	M	27	Posterior (combined)	7	C
27	M	49	Three-column	6	C
28	M	57	Three-column	7	C
29	F	51	Three-column	10	C
30	M	42	Posterior (combined)	7	C
31	F	46	Posterior (combined)	7	C
32	F	39	Posterior (combined)	7	C
33	M	44	Medial (combined)	7	C
34	M	47	Posterior (combined)	7	C
35	M	40	Three-column	10	C
36	M	65	Posterior (combined)	7	C
37	F	36	Three-column	7	C
38	M	59	Medial (combined)	6	C
39	M	64	Posterior (combined)	7	C
40	F	66	Three-column	7	C
41	F	57	Posterior (combined)	8	C
42	M	50	Posterior (combined)	7	C
43	M	51	Three-column	6	C
44	F	43	Posterior (combined)	7	C

M, male; F, female; O, observation; C, control.

**FIGURE 1:**
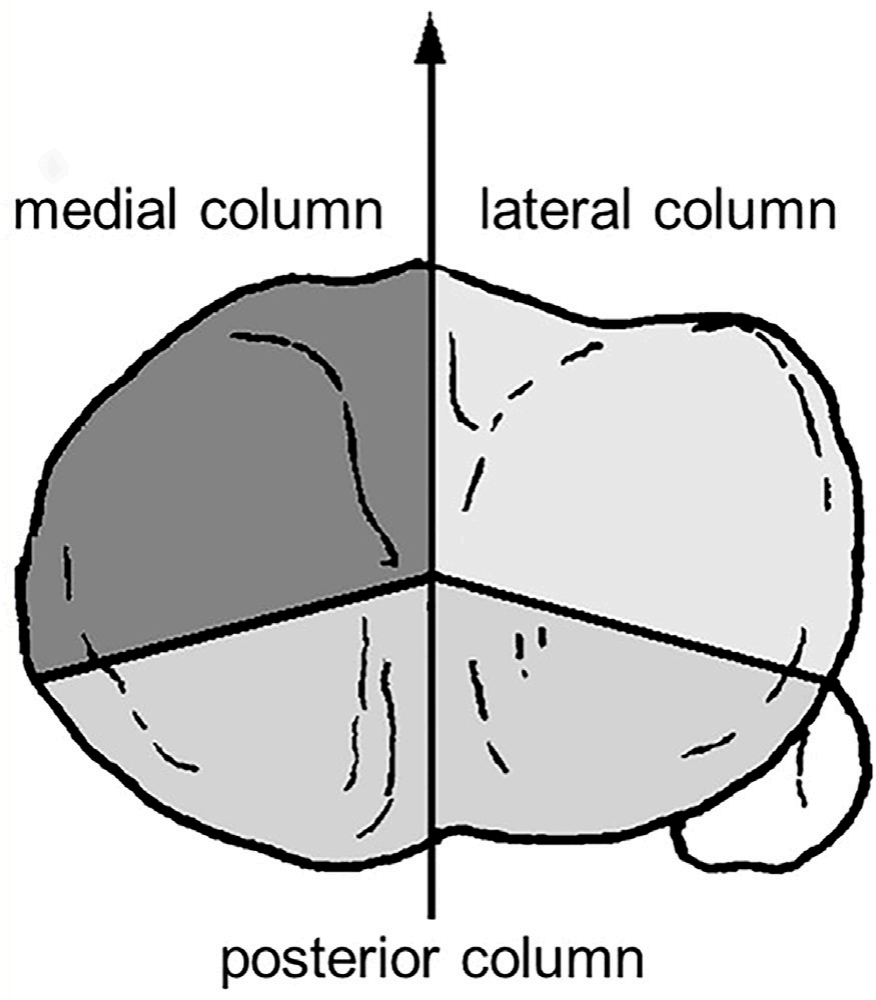
Schematic diagram of the three-column classification for tibial plateau fractures.

All 19 cases were unilateral closed fractures and classified as per the three-column classification system for tibial plateau fracture (Luo et al., 2010) (Figure 1), including 7 cases of simple posterior column fractures, 3 cases of posteromedial column fractures, 4 cases of posterolateral column fractures, and 5 cases of three-column fractures. Causes of injury included high fall accidents in 3 cases, Traffic accidents in 11 cases, and others in 5 cases.

All 25 cases were unilateral closed fractures, including 1 case of simple posterolateral column fracture, 4 cases of combined medial column fracture, 12 cases of combined anterolateral column fracture, and 8 cases of three-column fractures. Causes of injury included high fall accidents in 5 cases, Traffic accidents in 14 cases, and others in 6 cases.

### 2.2 Inclusion and exclusion criteria

Inclusion criteria included a clear diagnosis of tibial plateau fracture and conforming to the classification of posterior tibial plateau fracture; patients who underwent a CT scan of the affected knee for definite diagnosis or condition assessment; and patients who underwent at least 6 months of follow-up time and had complete relevant data.

Exclusion criteria included conservative treatment for various reasons; CT and MRI examinations showed diseases that were not limited by the knee joint itself, such as rheumatoid arthritis; and patients with other serious medical system diseases or end-stage tumors.

### 2.3 Preoperative preparation

Routine examinations on admission included routine blood, urine, and stool analyses, coagulation, liver and renal function, ion biochemistry, immune series, and inflammatory marker tests, and physical investigations comprising electrocardiogram (ECG), chest radiograph, cardiac ultrasound, ultrasound of both lower limb veins (for thrombosis), pulmonary function, anterolateral X-rays of the affected knee, CT scan, and 3D reconstruction of the knee (to clarify the fracture and determine the fracture type), and MRI of the knee (to determine whether there was damage to the meniscus and ligaments).

Preoperative assessments included sensation and movement of the lateral calf and foot, and preliminary assessment of neurovascular injuries. The affected knee was immobilized using plaster at approximately 30^o^ knee flexion with lower limb pads assisting in elevation to reduce swelling, followed by cold compresses. An appropriate amount of mannitol was injected to promote swelling of the affected limb. Patients were also advised to move their toes moderately to promote blood circulation and were given oral non-steroidal anti-inflammatory drugs and weak opioids for pain relief. Patients with combined non-skeletal muscular system injuries were referred to the relevant departments for assistance and were operated on when their condition was stable and they could tolerate surgery.

For patients with underlying medical diseases, the relevant departments were consulted to help manage the disease. For patients with hypertension, preoperative blood pressure was controlled below 150/100 mmHg. For diabetic patients, preoperative blood glucose was monitored, and symptomatic treatment was given to control glucose below 10 mmol/L. Surgery was performed after dermographia appeared in the swollen knee joint and soft tissue conditions improved.

Patients routinely fasted from food and water before surgery, and levofloxacin or cefoperazone sulbactam sodium (1 g) was administered intravenously 30 min before surgery to prevent infection. Preoperative blood preparation was performed to prevent life-threatening blood loss.

### 2.4 Modified posterolateral approach

**FIGURE 2:**
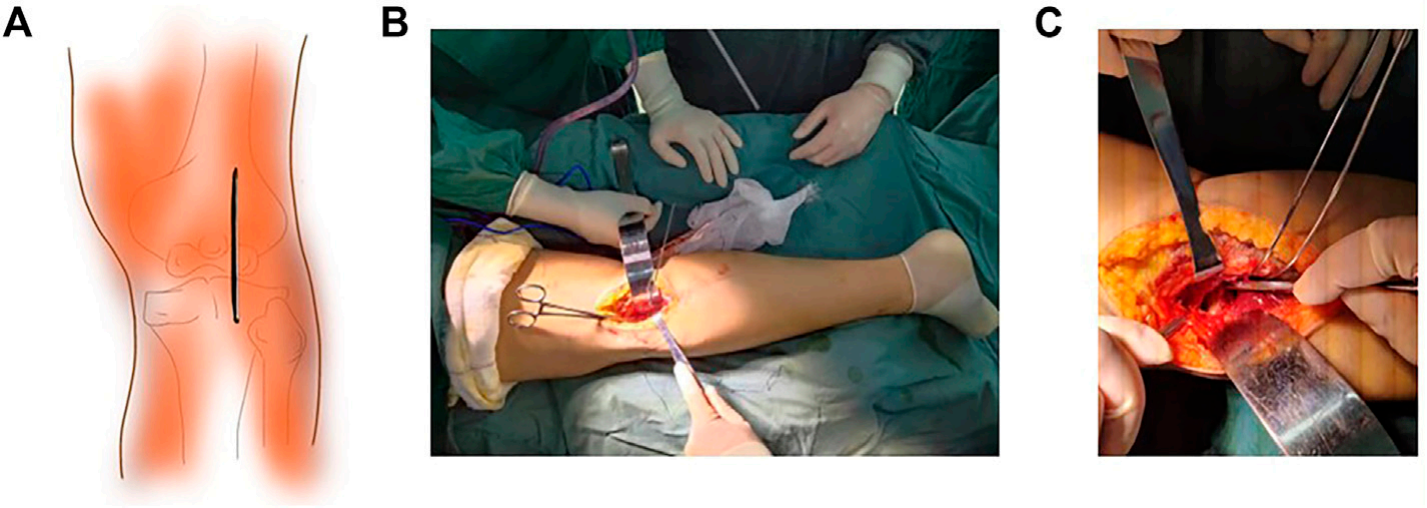
Schematic and intraoperative view of the modified lateral approach. **(A)** Schematic diagram of the modified posterolateral approach incision. **(B)** Exposure of the posterolateral tibial plateau. **(C)** The posterolateral joint surface of the tibial plateau under direct vision.

Combined spinal-epidural anesthesia or general anesthesia was given. Patients were in the prone position with padding under the affected knee joint and keeping the knee joint in a straight position. Using the fibular head as a landmark, a straight incision of 6–8 cm was made—proximal to distal—from 3 cm medial to 3 cm above the knee joint line (Figure 2A). The superficial tissue was incised medially in the biceps femoris muscle. The common peroneal nerve was bluntly stripped and pulled laterally with adhesive tape to protect the common peroneal nerve. The lateral collateral ligament was pulled laterally and the lateral head of the gastrocnemius muscle was pulled medially. During this process, the anterior tibial artery and the lateral inferior knee artery should be protected with care and the lateral inferior knee artery could be ligated if necessary. The popliteal muscle was retracted laterally and inferiorly (sometimes the popliteal muscle was cut) and the joint capsule was dissected to expose the posterolateral tibial plateau (Figure 2B). The lateral meniscus was lifted upward, to view the posterolateral joint surface of the tibial plateau under direct vision (Figure 2C).

**FIGURE 3:**
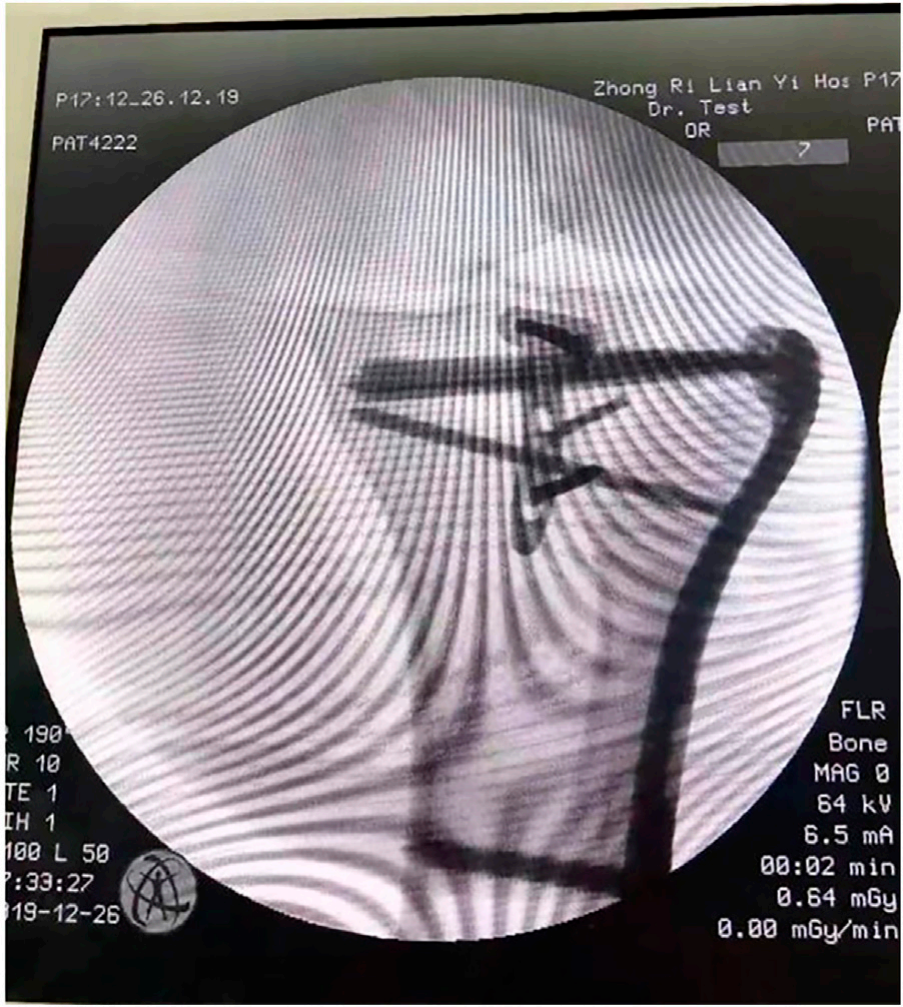
Intraoperative C-arm fluoroscopy to confirm fracture reduction.

The fracture was temporarily fixed with Kirschner needles and observed by the naked eye and C-arm fluoroscopy (Figure 3). If fracture reduction was satisfactory, a steel plate was grafted. Occasionally, additional screws were used to fix fracture fragments of the posterior wall. In cases of joint surface collapse, bone grafting was used to fill in the fracture after reduction.

### 2.5 Postoperative management

In both the observation and control groups, the affected limb was elevated using a lower limb pad to reduce swelling, while passive flexion and extension exercises with the aid of adjustable knee braces were initiated on day 2 postoperatively. Generally, the knee joint could be flexed up to 90° at 2 weeks postoperatively, and the affected limb was partially weight-bearing at 6 weeks postoperatively and fully weight-bearing at 3 months postoperatively if bony healing was confirmed by X-ray films.

### 2.6 Outcome measures and statistical validation

Significant differences among the two groups were observed the duration of the operation, intraoperative bleeding, postoperative joint surface collapse loss, and knee function scores. X-rays were taken immediately, at 6 weeks, 12 weeks, 6 months, and 1 year postoperatively. The distance from the highest point of the fibular head to the joint surface of the tibial plateau was measured by CT at various times after surgery to assess the severity of joint surface collapse. Knee active range of motion was measured using a standard protractor. The Hospital for Special Surgery (HSS) and Lysholm functional scales were employed for assessing the knee joint function. The HSS score is a 100-point scale that consists of six parts: pain, function, movement range, muscle power, knee flexion deformity, and stability. The Lysholm score also ranges from 0 to 100 and involves eight aspects: limp, pain, support, interlocking, swelling, instability, stair climbing, and squatting. The score was proportional to the total score. Knee joint function is proportional to the HSS and Lysholm scores. In this study, the total HSS and Lysholm scores were derived from the patient’s self-assessment score of each item at the telephone follow-up or outpatient follow-up visit.

SPSS 25.0 software was used for statistical analyses. Normally distributed data were expressed as mean ± standard deviation (SD) and the independent samples *t*-test was used for comparisons between groups. Non-normally distributed data were expressed as median (quartiles) and intergroup comparisons were made using the non-parametric rank sum test. A value of *p* < 0.05 was considered to be significant.

## 3 Results

A 12-month follow-up was completed in all 44 cases. All patients in the observation and control groups had one-stage healing of the surgical incision without incisional infection or skin necrosis. In the observation group, one patient with common peroneal nerve symptoms recovered 1 month after surgery, and there was no accidental injury to the anterior tibial vessels or the lateral inferior knee artery. Postoperative radiographs showed good fracture reduction, no joint surface collapse or loss, no loss of fracture reduction or breakage of internal fixation, and no internal or external knee deformity. All patients achieved fracture healing within 8–16 weeks after surgery.

### 3.1 Comparison of baseline and immediate postoperative conditions of patients between the two groups

**FIGURE 4:**
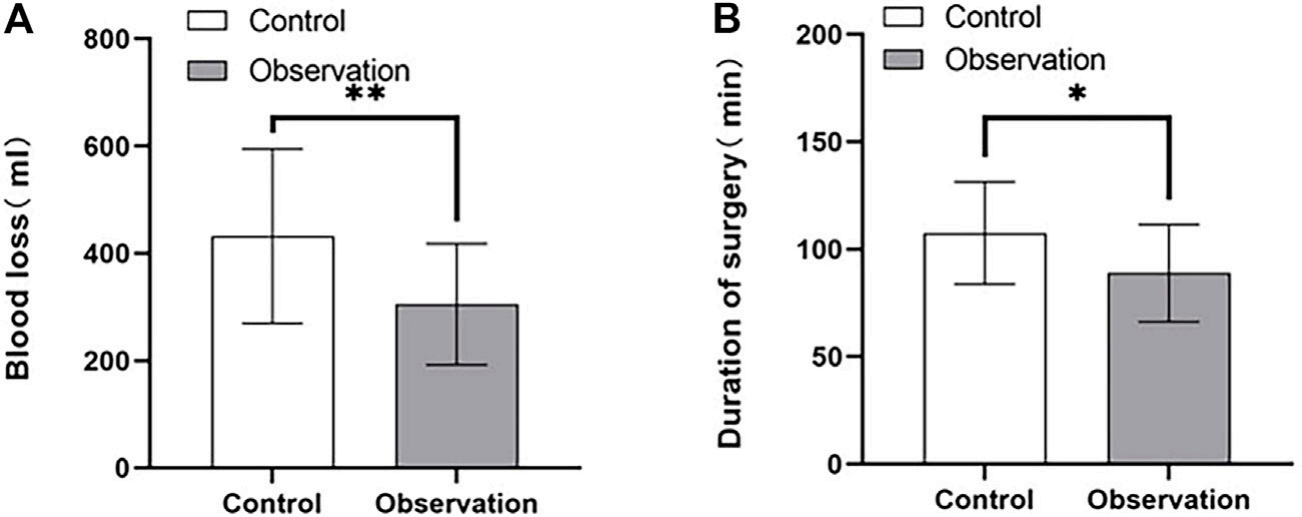
Comparison of intraoperative bleeding **(A)** and operative time **(B)** between control and observation groups.*p < 0.05, **p < 0.01.

The baseline and immediate postoperative conditions of the patients in both groups are shown in Table 2. There was no statistically significant difference in sex, age, and preoperative preparation time between the two groups (*p* > 0.05). The amount of blood loss (Figure 4A) and operative time (Figure 4B) in the observation group were significantly less than those in the control group (*p* < 0.01 and *p* < 0.05, respectively).

**TABLE 2:** Intergroup comparison of patients’ conditions at baseline and immediately after surgery.

Parameter	Group		Z/t	*p*
	Observation (*n* = 19)	Control (*n* = 25)		
Sex (M/F)	13/6	14/9	−1.109	0.762
Age (years)	44.00 ± 10.47	47.80 ± 10.27	0.004	0.274
Preoperative preparation time (day)	7 (7–8)	7 (7–7)	−0.812	0.417
Bleeding (mL)	305.26 ± 112.90	432.00 ± 162.58	−2.904	0.006
Operative time (min)	88.95 ± 22.58	107.60 ± 23.76	−2.634	0.012

M: male; F: female.

### 3.2 Comparison of the depth of tibial plateau collapse between the two groups

**FIGURE 5:**
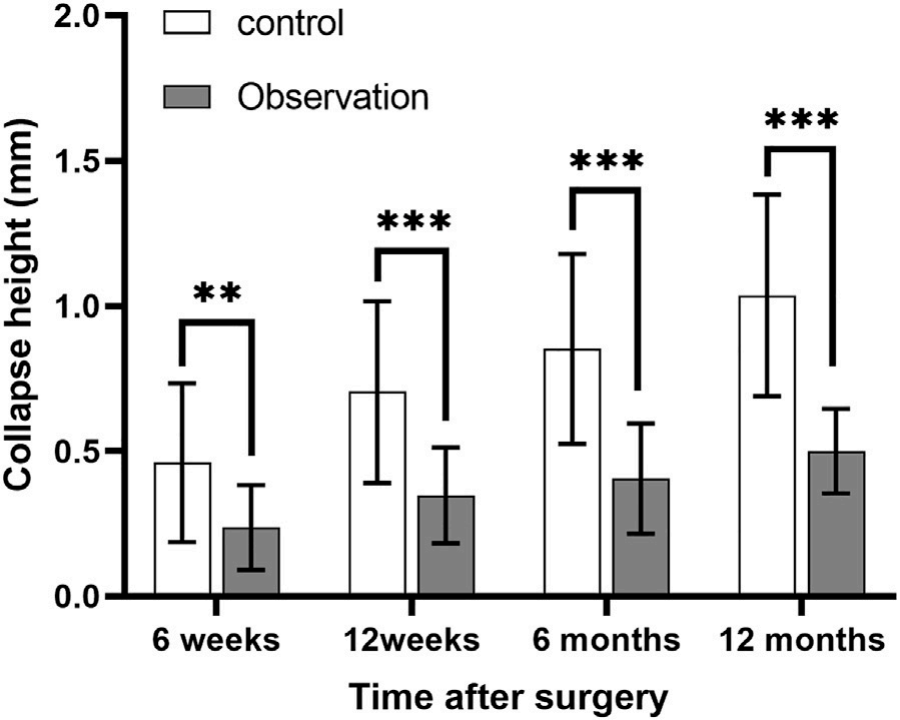
Comparison of the depth of tibial plateau collapse between the control and observation groups. **p < 0.01, ***p < 0.001.

Data on the depth of joint surface collapse in the patients after surgery conformed to a normal distribution. Significant differences existed between the two groups, as indicated by independent samples *t*-test (Table 3). At 6 weeks, 12 weeks, 6 months, and 12 months following surgery, the observation group’s tibial plateau collapse was much less severe than that of the control group (Figure 5).

**TABLE 3:** Intergroup comparison of the depth of tibial plateau collapse.

Time post-surgery	Group		t	*p*
	Observation (*n* = 19)	Control (*n* = 25)		
6 weeks	0.26 ± 0.11	0.46 ± 0.27	3.230	0.003
12 weeks	0.34 ± 0.16	0.70 ± 0.31	4.509	0.000
6 months	0.40 ± 0.18	0.85 ± 0.32	5.308	0.000
12 months	0.50 ± 0.14	1.03 ± 0.34	6.954	0.000

Data are expressed as mean ± SD.

### 3.3 Intergroup comparison of fracture healing 12 months after surgery

**FIGURE 6:**
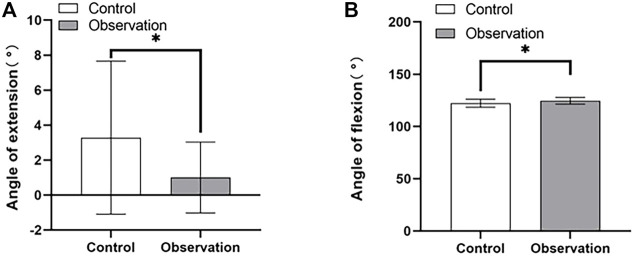
Comparison of postoperative knee flexion and extension function between the control and observation groups. **(A)** Knee extension angle. **(B)** Knee flexion angle. *p < 0.05.

**FIGURE 7:**
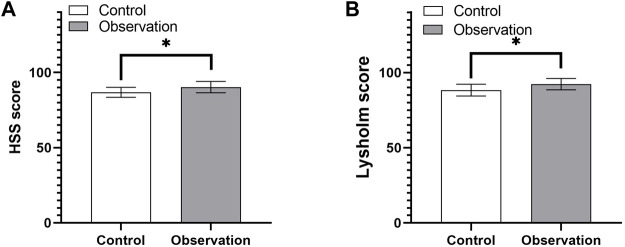
Comparison of the HSS **(A)** and Lysholm **(B)** scores between the control and observation groups. **p < 0.01. HSS: Hospital for Special Surgery.

In the observation group, the HSS knee function score at the final follow-up was excellent in 18 cases and good in 1 case. In the control group, the score was excellent in 20 cases and good in 5 cases. There were differences in knee flexion and extension, HSS score, and Lysholm score between the two groups, while there was no significant difference in the rear camber of the medial and posterior plateau of the tibia (Table 4). Postoperative knee flexion and extension recovered better in the observation group, suggesting that the modified posterolateral approach is more beneficial to the recovery of postoperative knee function (Figures 6A, B). The postoperative knee HSS and Lysholm scores suggesting that the modified posterolateral approach could reduce postoperative complications and aid in the recovery of Knee Mobility (Figures 7A, B).

**TABLE 4:** Intergroup comparison of fracture healing of the knee joint 12 months after surgery.

Outcome measure	Group		Z/t	*p*
	Observation (*n* = 19)	Control (*n* = 25)		
Posterior slope of the medial tibial plateau (^o^)	9.71 ± 1.92	9.66 ± 1.81	0.073	0.092
Posterior slope of the posterior tibial plateau (^o^)	9.77 ± 1.81	9.71 ± 1.78	0.110	0.913
Flexion (^o^)	124.63 ± 3.27	122.24 ± 3.86	2.170	0.036
Extension (^o^)	0 (0–0)	0 (0–5)	−1.975	0.048
HSS score	90.32 ± 3.74	86.92 ± 3.40	3.141	0.003
Lysholm score	92.26 ± 3.81	88.28 ± 3.92	3.377	0.002

HSS, hospital for special surgery.

### 3.4 Typical case

**FIGURE 8:**
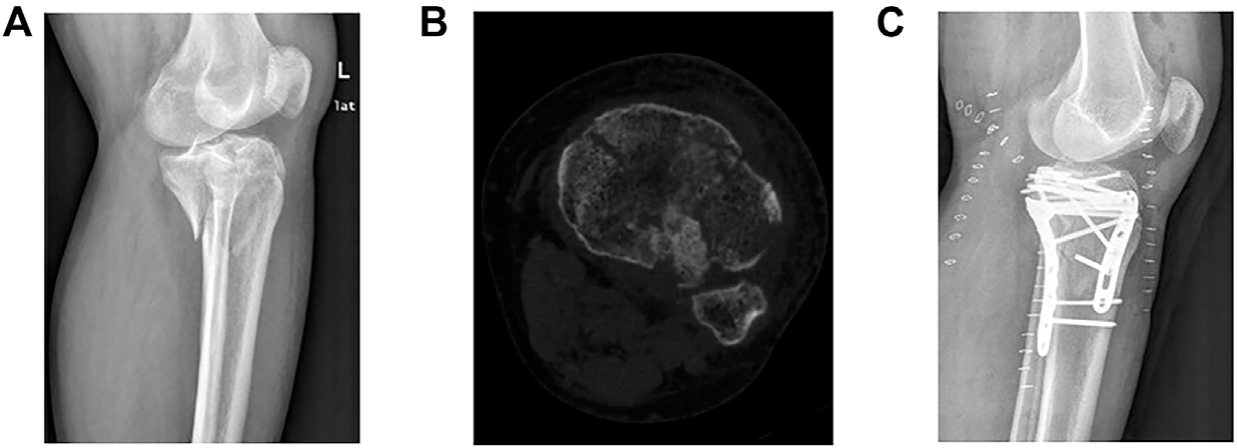
Imaging examinations of the affected limb before and after surgery. **(A)** Preoperative lateral X-ray of the affected limb, showing posterolateral fracture fragments. **(B)** Transverse CT of the affected limb, showing fractures of the lateral, medial, and posterior columns. **(C)** Postoperative lateral X-ray of the affected limb, showing good joint surface reduction and fracture fixation.

A 47-year-old male was hospitalized for pain and impaired movement of the left knee 6 h after a Traffic accident. His left knee joint presented with mild swelling, pressure pain, and limited range of flexion. Radiological findings revealed a broken left tibial plateau with separation and displacement of the condyles and joint surface collapse. The patient was diagnosed with a comminuted fracture of the left tibial plateau. A preoperative lateral X-ray of the affected limb revealed posterolateral fracture fragments (Figure 8A). Transverse CT of the affected limb showed lateral column, medial column, and posterior column fractures (Figure 8B), and the fracture type was determined as a three-column fracture according to the three-column fracture classification system. Swelling of the affected limb subsided 7 days after the injury, and the left tibial plateau fracture was exposed *via* the posterolateral incision approach. Intraoperatively, the lateral plateau was separated and displaced, the fracture fragment was split, and the posterolateral joint surface had collapsed. After fracture reduction, a homogeneous allograft bone graft was performed (the amount of bone graft was approximately 10 g). The posterolateral joint surface was fixed with a T-plate, and the fracture mass of the articular surface of the medial and lateral columns, which was large and mildly displaced backward, was fixed with a reconstruction plate (Figure 8C).

**FIGURE 9:**
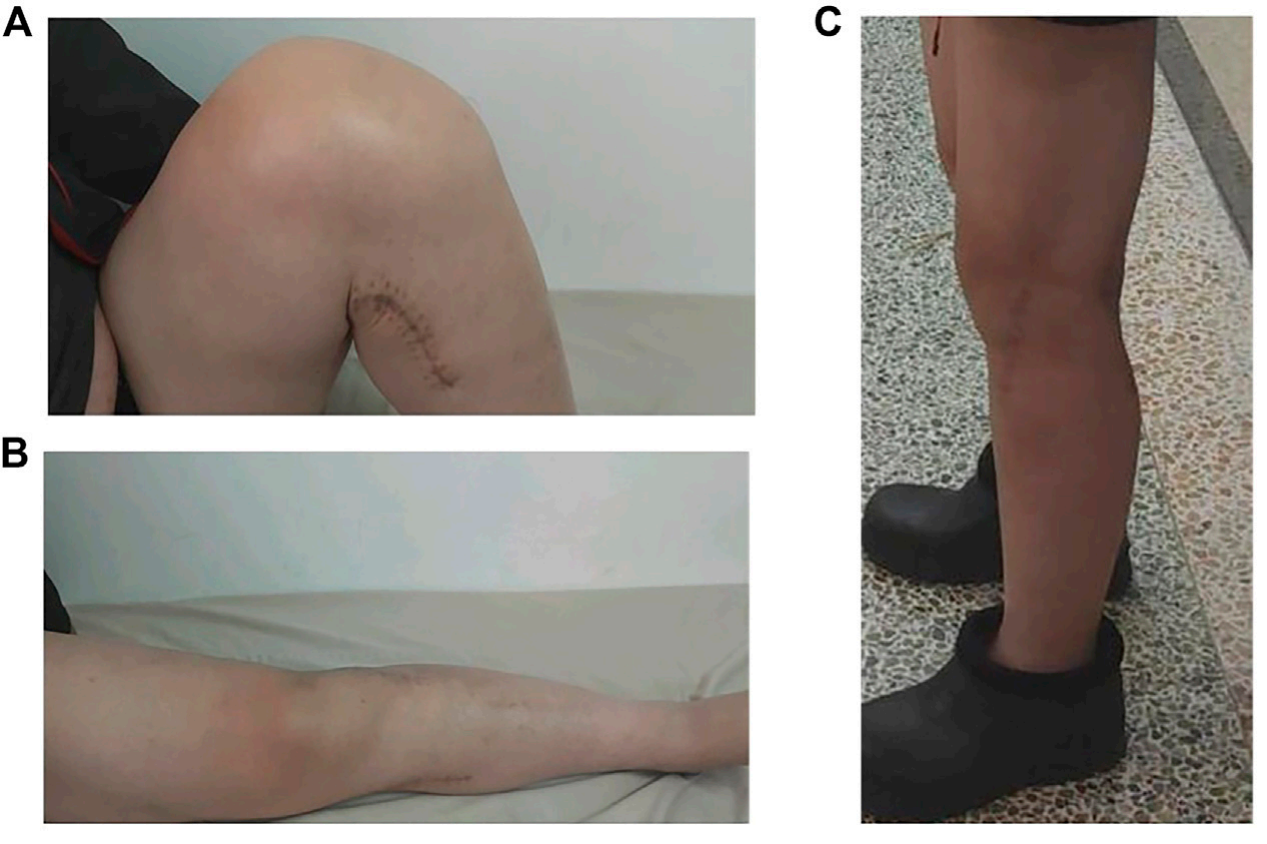
Functional recovery of the knee joint 8 months postoperatively Flexion **(A)** and extension **(B)** of the affected limb, and full weight-bearing walking **(C)**.

The patient was guided to perform quadriceps-stretching exercises and functional exercises of the joint 3 days after surgery. The joint flexion and extension range was close to 90° at 10 days after surgery. During the 12-month follow-up, the fracture healed at 6 months postoperatively, and the flexion and extension function was good at 8 months (Figures 9A, B), with full weight-bearing walking (Figure 9C) and an excellent HSS score.

## 4 Discussion

Appropriate clinical measures for tibial plateau fractures can improve the functional recovery of the lower limbs and lessen the likelihood of problems following surgery. For posterolateral plateau fractures, different surgical approaches have different outcomes for recovery of knee function of complex tibial plateau fractures (Xu et al., 2013; Boluda-Mengod et al., 2021; Krause and Frosch, 2022).

The use of horizontal rafting plates *via* the conventional anterolateral approach can reconstruct and support posterolateral fractures and the reconstruction plate can be supported *via* a medial approach (Bermúdez et al., 2008). The modified anterolateral approach can provide adequate exposure of fracture fragments (Cho et al., 2016). Lateral femoral epicondylar osteotomy has also been used to adequately access the tibial plateau without damage to soft tissue structures (Yoon et al., 2015). The use of horizontal rafting plates *via* the conventional anterolateral approach can reconstruct and support posterolateral fractures, and place a steel plate to assist in supporting the lateral plate for bone remodeling.

The posterolateral approach was proposed by Lobenhoffer et al. (1997), and could be used alone or in combination with a fibular osteotomy. Subsequently, Yu et al. (2010) proposed a fibular head osteotomy to exposure of the posterior lateral tibial plateau. A posterolateral transfibular neck approach has also been proposed as a treatment measure, in which the fibular head is upturned to fully expose the posterolateral aspect of the tibial plateau; however, the likelihood of peroneal nerve palsy rising (Solomon et al., 2010). Tao et al. (2008); Luo et al. (2010); He et al. (2013) introduced a modified reversed L-shaped approach for posterolateral tibial plateau fractures, which can provide a better operative field for complex posterolateral tibial plateau fractures. However, care is required to dissect and protect the common peroneal nerve (Chang et al., 2014). Moreover, the distal end should extend less than 5 cm from the joint line, thereby avoiding an injury to the inferolateral knee branch of the popliteal artery and this approach has been used less frequently in recent years for purely isolated posterolateral tibial plateau fractures (Sun et al., 2013). Frosch et al. (2010) proposed a posterolateral approach that allowed for good visual control of the joint surface, but exposure of the surgical field was poor. To address the problem of poor joint surface exposure with the posterolateral approach, Carlson et al. (Carlson, 2005) designed a posterolateral S-curve approach, which is a good choice for visual field exposure but results in greater soft tissue damage.

Based on the anatomical observations and clinical applications in this study, we found that the modified posterolateral approach provides good exposure of the posterior aspect of the tibial plateau and satisfactory fracture reduction in most cases of posterior tibial plateau fractures. The traditional approach uses a lateral position; we use a prone position. Therefore, compared to other postero-lateral approaches, our approach has a smaller incision that allows for a more medial approach to avoid overstretching the common peroneal nerve. Intraoperative bleeding and operative time with the modified posterolateral approach are less than those with the traditional lateral approach. This indicates that the modified posterolateral approach achieves surgical field exposure with less damage and shorter time and has good postoperative results in preventing joint surface collapse. In addition, with the development of internal fixation devices, the 3.5-mm T-plate can be placed smoothly over the posterior aspect of the tibial plateau *via* the posterolateral approach, and its proximal row of screws can be placed under the posterior joint surface of the tibial plateau, forming a raft-like support for the joint surface. In some patients with posterolateral wall split fractures, which are difficult to reduce through a posterolateral incision, the fracture can be exposed through a modified posterolateral incision and the posterolateral placement of a 3.5-mm T-plate for internal fixation is relatively easy, avoiding the extension of a posterolateral incision and reducing soft tissue injury.

Tibial plateau collapse can cause knee pain, limit knee function, and even require surgery in severe cases (if the depth of joint surface collapse exceeds 2 mm). As demonstrated in the typical case, the comminuted fracture resulted in severe collapse of the articular surface, and the fracture and knee function recovered well after treatment with a modified posterior lateral approach. In this study, the depth of tibial plateau collapse in patients treated with the modified posterolateral approach was significantly less compared with that of the conventional lateral approach at 6 weeks, 12 weeks, 6 months, and 12 months postoperatively. This suggests that the modified posterolateral approach can prevent postoperative joint surface collapse of the tibial plateau in patients with tibial plateau fractures. The knee flexion and extension function, HSS score, and Lysholm score at 12 months after surgery revealed that the modified posterior lateral approach was more helpful for the postoperative recovery of knee function and reduced the occurrence of postoperative complications compared with the conventional lateral approach.

In conclusion, this new surgical approach with posterolateral plate fixation has the following advantages: 1) the approach can strengthen and prevent collapse of the tibial plateau joint surface; 2) the posterior joint surface of the plateau can be viewed directly through a smaller incision; 3) most fractures can be reduced and fixed through a simple posterolateral approach, with good functional recovery of the knee joint; 4) bleeding, operative time and the risk of injury to the anterior tibial vessels are all reduced compared with other posterolateral approaches; 5) the approach can be used for isolated posterior fractures and posterolateral fractures of the tibial plateau. However, the approach does have some limitations: 1) when fracture reduction is conducted *via* the posterolateral space, the common peroneal nerve that requires exposure is at risk of being injured; 2) in some patients, the popliteal muscle an important stabilizing structure of the posterolateral knee—is difficult to retract proximally, requiring a hamstringotomy to expose the fracture. Although popliteal dissection can be repaired with sutures, it is unclear whether early functional exercise and long-term knee stability can be achieved after popliteal dissection; 3) through a posterolateral incision, only the fracture in the posterior part of the Gerdy’s tubercle can be exposed anteriorly. However, it is difficult to expose a fracture that extends to the anterior part of the Gerdy’s tubercle. Finally, the limited sample size in this study means that further research is needed to draw more experience in clinical practice.

## 5 Conclusion

The modified posterolateral approach for posterior tibial plateau fractures can effectively prevent joint surface collapse of the tibial plateau and promote the recovery of knee function in patients. The approach also has few postoperative complications and good clinical efficacy, and is worth promoting in clinical practice.

## Data Availability

The original contributions presented in the study are included in the article/supplementary material, further inquiries can be directed to the corresponding author.
